# The Cardiovascular Stress Response as Early Life Marker of Cardiovascular Health: Applications in Population-Based Pediatric Studies—A Narrative Review

**DOI:** 10.1007/s00246-020-02436-6

**Published:** 2020-09-02

**Authors:** Meddy N. Bongers-Karmaoui, Vincent W. V. Jaddoe, Arno A. W. Roest, Romy Gaillard

**Affiliations:** 1 The Generation R Study Group, Erasmus MC, University Medical Center, PO Box 2040, 3000 CA Rotterdam, The Netherlands; 2 Department of Pediatrics, Erasmus MC, University Medical Center, Rotterdam, The Netherlands; 3 Department of Pediatrics, Leiden University Medical Center, Leiden, The Netherlands

**Keywords:** Epidemiology, Pediatric cardiology, Exercise, MRI

## Abstract

Stress inducement by physical exercise requires major cardiovascular adaptations in both adults and children to maintain an adequate perfusion of the body. As physical exercise causes a stress situation for the cardiovascular system, cardiovascular exercise stress tests are widely used in clinical practice to reveal subtle cardiovascular pathology in adult and childhood populations with cardiac and cardiovascular diseases. Recently, evidence from small studies suggests that the cardiovascular stress response can also be used within research settings to provide novel insights on subtle differences in cardiovascular health in non-diseased adults and children, as even among healthy populations an abnormal response to physical exercise is associated with an increased risk of cardiovascular diseases. This narrative review is specifically focused on the possibilities of using the cardiovascular stress response to exercise combined with advanced imaging techniques in pediatric population-based studies focused on the early origins of cardiovascular diseases. We discuss the physiology of the cardiovascular stress response to exercise, the type of physical exercise used to induce the cardiovascular stress response in combination with advanced imaging techniques, the obtained measurements with advanced imaging techniques during the cardiovascular exercise stress test and their associations with cardiovascular health outcomes. Finally, we discuss the potential for cardiovascular exercise stress tests to use in pediatric population-based studies focused on the early origins of cardiovascular diseases.

## Introduction

Cardiovascular diseases are a major public health problem worldwide [1]. Because of the large clinical impact that cardiovascular diseases have in adulthood, most research has focused on adult populations. Accumulating evidence suggests that cardiovascular diseases may at least partly originate in the earliest phase of life [2, 3]. Adverse exposures acting at different stages of fetal and early postnatal development, may lead to permanent adaptations in the structure, physiology and function of cardiovascular organ systems, predisposing to an increased risk of cardiovascular risk factors in childhood and cardiovascular disease in later life [4–7]. It is well-known that cardiovascular risk factors, such obesity and a higher blood pressure, often track from childhood into adulthood and are associated with cardiovascular diseases in later life [8, 9]. These effects are even stronger among individuals within an unhealthy lifestyle as adults.[10] Multiple observational studies have shown associations of adverse maternal, placental and fetal exposures during pregnancy with an impaired cardiovascular development in the offspring in both childhood and adulthood [2, 3, 11]. However, despite these observed associations, the underlying mechanisms remain unclear and it remains challenging to identify children at higher risk of cardiovascular diseases in later life who may especially benefit from early interventions.

Among pediatric populations, exercise testing of the cardiovascular system may be used as a novel method to detect subtle differences in cardiovascular development and to better identify children at risk of reduced cardiovascular health in later life. Physical exercise causes a stress situation for the cardiovascular system and requires important circulatory adaptations to maintain an adequate perfusion of the body. Already, cardiovascular exercise stress testing is widely used in clinical practice to reveal subtle pathology in adult and pediatric diseased populations [12–15]. In adult populations with cardiac abnormalities and cardiovascular diseases, an abnormal response of the cardiovascular system to exercise is associated with further deterioration of cardiovascular diseases and an increased risk of mortality [16, 17]. In pediatric populations, cardiovascular exercise testing is used especially in children with congenital heart diseases, but also with Kawasaki disease, arrhythmias, acquired valvular heart disease, cardiomyopathy and hypertension to evaluate the severity of the condition, to assess the effects of pharmacological or surgical treatment or to induce and detect arrhythmias [15, 18, 19]. Also among these pediatric patients, an abnormal cardiovascular response to exercise is associated with poorer cardiovascular health outcomes, reduced exercise capacity and overall reduced quality of life [20]. Recently, evidence from small studies among pediatric populations without cardiovascular pathology suggests that the cardiovascular exercise stress test may provide important information on cardiovascular health in non-diseased pediatric populations [21, 22]. This underlines the importance of obtaining a better understanding of the potential use of the cardiovascular exercise stress test in pediatric populations in both research and clinical settings to identify children with an impaired cardiovascular health profile. In this narrative review, we discuss the potential for assessment of the cardiovascular stress response to exercise in pediatric population research. We discuss the physiological cardiovascular stress response, the use of different exercise methods and advanced imaging techniques to measure the cardiovascular stress response and the potential of using the cardiovascular stress response for future pediatric population research focused on the early origin of cardiovascular diseases. This review is partly based on two Medline searches (through PubMed) up to January 2019 in order to identify relevant studies focused on the use of isometric handgrip exercise to induce the cardiovascular stress response in children and its use in combination with cardiac Magnetic Resonance Imaging (cMRI) scanning. The used search terms are described in Textbox 1.”

**Textbox 1:**
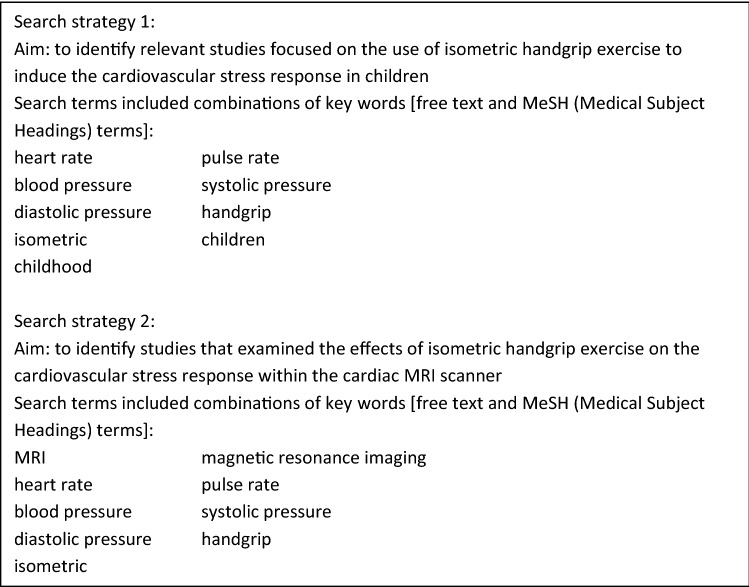
Used search strategies for this narrative review

## Cardiovascular Stress Response to Exercise

**Fig. 1:**
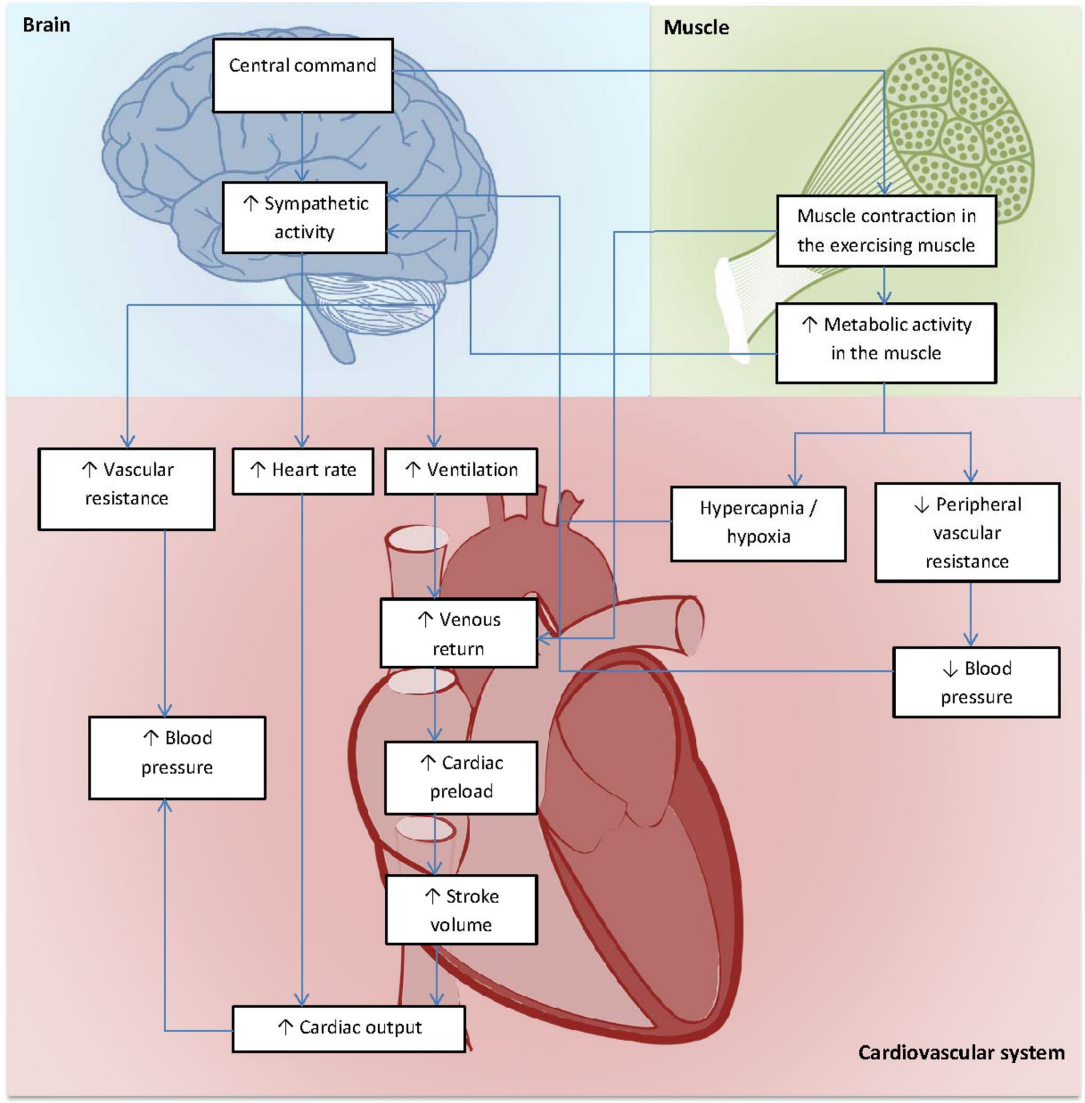
Figure of the cardiovascular stress response, focusing on the main effects in the exercising muscles, brain and the cardiovascular system

One of the most well-known stressors of the cardiovascular system is physical exercise, which leads to multiple adaptations in the cardiovascular system. An overview of the cardiovascular stress response is given in Fig. 1. During exercise, muscle activity increases the demand for oxygen. The response of the circulatory system is designed to match these higher oxygen requirements and thus higher blood flow in the exercising muscles. The cardiovascular response to exercise consists of a rise in heart rate, heart contractility and blood pressure [23]. Due to the mechanical skeletal muscle pump and exaggerated movement of the respiratory pump, exercise leads to a higher venous return, which will subsequently lead to an increased stroke volume. Both increases in heart rate and stroke volume lead to a higher cardiac output (CO). Because of the increase in CO and increasing vascular resistance in the abdominal viscera and non-active skeletal muscles, blood pressure will increase [24–30]. There are several underlying autonomic mechanisms responsible for the sympathetic activation that causes the cardiovascular response on exercise, including corticohypothalamic pathways and peripheral reflexes [28, 31–33]. To enable these extensive physical adaptations to exercise, a healthy cardiovascular system is needed. Adaptations to physical exercise may not only be impaired in clinical populations with known cardiovascular or cardiac disease. Already, when subtle subclinical differences in cardiovascular health are present, this may lead to suboptimal adaptions of the cardiovascular system to the increased demands induced by exercise [34, 35]. Thus, measurement of the cardiovascular stress response to physical exercise may reveal subtle pathology that would have been undetectable at rest in research settings with presumably healthy pediatric populations.

## Measurements of the Cardiovascular Stress Response in Pediatric Population Studies

Ideally, multiple cardiovascular measurements are obtained during rest, exercise and recovery to obtain an adequate evaluation of the response of the cardiovascular system to exercise. These measurements include heart rate response and recovery, oxygen saturation changes, blood pressure response and recovery and changes in stroke volume, ejection fraction and cardiac output.

Clearly, heart rate, oxygen saturation, electrocardiography and blood pressure response and recovery, are most easily obtained. Previous studies have mainly focused on these measurements to determine an abnormal cardiovascular response to physical exercise [15, 19, 36]. Abnormal cardiovascular response to exercise include an abnormal chronotropic response, abnormal heart rate recovery response, excessive rises in exercise blood pressure and exercise hypotension [23]. An abnormal chronotropic response is the inability of the heart rate to increase equivalent to the increasing demand of blood flow during exercise [37]. The inability to increase heart rate linearly in proportion to the physical effort, is common in both children and adults with congenital heart diseases and is associated with a poor prognosis[38–40]. An abnormal heart rate recovery response is usually defined as a decline in heart rate of ≤ 12 beats from peak exercise to one minute after cessation of the exercise test [23]. An excessive rise in exercise blood pressure is defined as a systolic blood pressure value exceeding the 95th percentile for exercise blood pressure [41, 42]. Exercise induced hypotension (EIH) can also occur, which is defined as a drop in systolic blood pressure during exercise below the pre-exercise value [43]. These impaired cardiovascular responses to exercise are strongly associated with cardiovascular events, diseases and mortality within adult populations, but smaller studies have also shown associations of an abnormal cardiovascular response to exercise with reduced cardiovascular health in children[12–14, 36, 44–51].

In addition to these common measures, there is an increasing awareness that advanced non-invasive cardiac imaging during exercise tests improves the value of the cardiovascular exercise tests as it allows detailed assessment of the structural and functional cardiac response to exercise [13, 52]. Non-invasive cardiac imaging modalities include echocardiography and the more advanced imaging modality of cardiac MRI scans. Exercise stress echocardiography is a commonly used imaging method to assess left ventricular function, wall motion, mitral valve function, pulmonary systolic pressure and diastolic function in response to exercise [42, 53–56]. In pediatric cardiology, stress echocardiography is mainly used in patients at risk for ischemic heart disease, such as children with Kawasaki disease, aortic stenosis, abnormal origin of the coronary arteries or children after coronary reimplantation [57, 58]. Echocardiography plays an important role in cardiac exercise testing due to its high imaging quality and ease of use. However, stress echocardiography has some important limitations. The dimensions of the right ventricle and stroke volume are challenging to assess. cMRI during exercise provides superior high resolution image quality and can produce 3D images of all the cardiac chambers, which allows for the most accurate and reproducible assessment of the cardiac response to exercise without geometric assumptions. cMRI also allows for assessment of the coronary artery system during exercise [59]. Although MRI has some limitations, such as the longer scan duration and higher costs, this more advanced imaging modality seems preferable in large population studies due to the superior reproducibility and detailed assessment of all cardiac chambers, which allows detection of small subclinical differences on a population level. Several small studies have used cMRI to obtain more detailed insight into cardiac adaptations to exercise and showed differences in cardiac response to exercise in diseased and non-diseased populations [60–62].

Thus, multiple measurements of the cardiovascular system are needed to fully address the cardiovascular stress response to exercise using both simple clinical measurements and advanced imaging techniques. Differences in these cardiovascular measurements are related to cardiovascular outcomes in later life in both adult and pediatric populations.

## Exercise Methods for Detailed Cardiovascular Stress Response Assessment in Pediatric Research

There are multiple methods available to induce the cardiovascular response to exercise. In clinical practice, the cardiovascular stress response is often tested by the use of pharmacological stressors such as adenosine or dobutamine [23, 63]. However, this method cannot easily be used in pediatric research settings and does not entirely compare to the cardiovascular exercise response to everyday exercise as in contrast to exercise induced cardiovascular stress, pharmacological stressors do not lead to an increased venous return and subsequent preload[52].

There are several ways to induce a cardiovascular stress response by physical exercise in pediatric populations, which can be used in combination with advanced imaging techniques to obtain a detailed measurement of the cardiovascular stress response. These different approaches include the use of a treadmill, bicycle and isometric handgrip exercise, each with its own exercise protocol and advantages and disadvantages. Table 1 gives a short description of each of the three exercise methods and briefly discusses its advantages and disadvantages based on studies and actual experience with different exercise methods of the authors. By using a treadmill, the subject performs a running exercise protocol. Most studies use the Bruce Treadmill Protocol to achieve peak stress [64]. After the exercise, the subject has to take place in the MRI scanner as quickly as possible to assess the detailed cardiac response to exercise. When the subject takes place in the MRI, new localizer scans are needed for correct cardiac scanning. This results in a time delay between peak stress and image acquisition that may allow the subject’s cardiovascular system to recover [64]. Another option is the use of vacuum mattress positioning devices in order to position the subject identically to the position in which the subject was positioned during the scans before the exercise was performed. However, this method is time consuming, worsens the claustrophobic feeling of the small space inside the MRI scanner and has to be extremely precise which can be challenging in pediatric studies. Contrary to the treadmill exercise, both the bicycle and isometric handgrip exercise can be performed within the MRI scanner, reducing the delay between the exercise and assessment of the cardiac response to the exercise[52, 65]. A bicycle test is performed with the use of MRI compatible foot pedals at the foot end of the MRI table. Just before scanning, the exercise is performed to high exertion, after which the subject stops the exercise and the cMRI scan is conducted [64]. Small studies among healthy volunteers have used different exercise protocols to achieve peak stress measured by a minimal heart rate or percentage of the maximal oxygen uptake [65, 66]. Ultra-fast and real-time scanning is required to limit the breath holding time. A long breath hold is not feasible after intensive exercise, especially in children. Only isometric handgrip exercise can be performed during cMRI scanning. In this exercise protocol, the subject squeezes the device at a maximal force to determine the maximum voluntary contraction (MVC). After a recovery period, the subject takes place inside the MRI scanner and takes the hand dynamometer in his or her dominant hand and squeezes the device at a certain percentage of the MVC for a certain period of time during the scan to induce the cardiovascular stress response to exercise [52]. This sustained handgrip method is eminently suited for pediatric research as this method is relatively easy to perform, does not lead to motion artifacts and can be performed during the scanning without the need for a real-time scan. Also, this exercise has the lowest costs in comparison with the other exercise methods.

**Table 1:** Description, advantages and disadvantages of three different types of exercise methods used to induce the cardiovascular stress response that can be used in combination with advanced imaging techniques

	Methods	Advantages	Disadvantages
Treadmill exercise	An MRI compatible treadmill is placed in the MRI room. After the exercise, the subject has to take place in the MRI scanner as quick as possible	– Exercise can be performed to maximum exertion [64] – Motion artifacts are less compared to dynamic exercise in a MRI device	– The time period between peak stress and image acquisition may allow the subject’s cardiovascular system to recover [52] – Ultra-fast scanning is required to limit the breath holding time. A long breath hold is not feasible after intensive exercise – The device has to be placed inside the MRI room to reduce the time delay
Bicycle exercise	An MRI compatible bicycle ergometer can be placed at the food end of the MRI table. Just before scanning, the exercise can be performed to high exertion. Then, the subject has to stop the exercise before the scan has started	– Exercise can be performed to high exertion. [65, 66] – Exercise inside the MRI device is possible	– Ultra-fast scanning is required to limit the breath holding time. A long breath hold is not feasible after intensive exercise – Scanning while exercising is not possible without any motion artifacts – A fully circular movement of the legs is not feasible due to the limited space in the MRI
Handgrip exercise	Immediately after the start of the exercise, the scan can be started. The exercise is performed during the scan protocol up to 8 min [52]	– Real-time scanning while exercising is possible without any motion artifacts [35] – Breath holds are feasible – Simple to implement and least expensive method – Good reproducibility [67]	– Exercise cannot be performed to maximum exertion

Thus, based on its advantages and disadvantages, we consider especially in large pediatric population-based cohort studies, handgrip exercise among the most feasible physical stressors to induce the cardiovascular stress response to exercise, as it is easy to perform for children and allows as only method real-time scanning without losing image quality due to movement artifacts. Although handgrip exercise cannot be performed to maximum exertion, many studies showed that isometric exercise significantly raises heart rate and blood pressure in children [68–79].

## Isometric Handgrip Exercise and the Effects on Heart Rate Variability and Blood Pressure in Pediatric Populations

The effects of isometric handgrip exercise on simple measurements of the cardiovascular stress response has been assessed by several studies in children both in the general population and in children at a higher risk of cardiovascular diseases. Table 2 summarizes the results and methods of the studies identified by our Medline search. In general pediatric populations, various handgrip exercise protocol haven been used. A study among 23 healthy children, aged 7–9 years examined the effects of 3 min at 30% MVC sustained handgrip on the cardiac index. The cardiac index was calculated by dividing the cardiac output (calculated as the product of heart rate and stroke volume) by body surface area (BSA). Stroke volume was calculated from the arterial pressure signal using the arterial pulse wave contour method. They found an increase of the cardiac index with 0.2L/min/m^2^ in response to isometric handgrip exercise [69]. A study among 217 children with a mean age of 13 years showed that a handgrip exercise of 2,5 min of sustained contraction at 30% MVC was associated with significant and clinically relevant changes in heart rate and blood pressure among boys and girls and that boys had greater systolic blood pressure responses than girls [73]. Among 162 healthy children with a mean age of 11 years it was shown that a sustained handgrip of 2 min at 60% MVC raises heart rate and blood pressure significantly [78]. Even handgrip exercises of only 30 s at 30% MVC and 4 min at 25% MVC have led to significant increases in blood pressure and heart rate in two other pediatric studies in 35 children with a mean age of 15 and 32 children with a mean age of 15 respectively [71, 77].

**Table 2:** Descriptive overview of studies examining the cardiovascular effects of isometric handgrip exercise in children

Name, year	Population	Used handgrip exercise protocol	Main cardiovascular outcomes
Dipla (2010) [68]	27 healthy boys: age: 11 years	3 min at 30% MVC	In rest obese boys had higher stroke volume and lower total peripheral resistance than lean boys. During exercise, ΔMAP was not significantly different between lean and obese boys (22.7 ± 2.6 vs. 19.6 ± 1.5 mmHg in lean vs. obese boys) ΔHR was higher in lean boys than in obese boys: 14.5 ± 1.6 vs. 8.2 ± 1.3BPM
Ferrara (1991) [78]	162 healthy children: age: 11 years	2 min at 60%MVC	Significant increase in BP and HR
Goulopoulou (2010) [69]	23 healthy children: age: 7–9 years	3 min at 30% MVC	SBP: 107.9 ± 2.0 mmHg to 122.1 ± 2.7 mmHG DBP: 64.6 ± 2.0 mmHg to 78.1 ± 2.5 nmmHG MAP: 82.8 ± 2.4mmHG to 96.4 ± 2.4 mmHg HR: 84.0 ± 2.0BPM to93.5 ± 2.2 BPM Cardiac index (L/min/m2): 1.5 ± 0.06 to 1.7 ± 0.07 Stroke index (mL/beat/m2: 17.6 ± 0.6 to 17.9 ± 0.7 Al rises were significant
Gumbiner (1983) [70]	18 healthy children 28 children with aortic insufficiency Age: 13 years	3 min at 33% MVC	Control: HR: 78 to 91 BPM (P < 0.05) Blood pressure 115/64 to 128/76 mmHg Patients: HR: 75.4 to 89.5 BPM (P < 0.05) Blood pressure: 117/53 to 150/72 mmHg
Laird (1979) [71]	32 healthy children: age: 15 years	4 min at 25%MVC	Heart rate (beats/min) 70 ± 9 to 88 ± 11 Systolic pressure (mm Hg): 110 ± 7 to 124 = 10 Diastolic pressure(mm Hg) 61 ± 8 to 76 ± 8 Mean pressure (mm Hg) 78 ± 7 to 92 ± 7 Al rises were significant
Legantis (2012) [72]	48 healthy children: age: 11.6 ± 0.3 years	3 min at 30% MVC	At rest and during exercise, unfit obese/overweight children had higher systolic, mean arterial pressure, and rate pressure product than fit obese/overweight children whose responses were similar to normal weight children, fit or unfit. Changes from rest, in cardiac output, cardiac index, and stroke volume were higher in unfit than in fit obese/overweight children
Matthews (1988) [80]	217 children: age: 13 years	2,5 min at 30% MVC	Significant increase in BP and HR which was larger in boys than in girls
Mehta (1996) [74]	18 children with presence of parental hypertension 29 healthy children Age: 10 to 18 years	4 min at 25% MVC	The between-group difference in heart rate was not statistically significant at rest (70 _ + 9 BPM vs 75 _ + 9 BPM) With exercise, the heart rates were significantly higher in subjects from the patients group (87 _ + 10 BPM vs 79—+ 13 PBM)
Nageswari (2007) [75]	20 obese/overweight children 20 non-obese children Age: 12–16 years	30%MVC until the point of fatigue	Change in diastolic BP: Control:15.9 ± 4.61mmHG Obese: 11.4 ± 4.02mmHG
Schieken (1983) [76]	264 students: age: 9–18 years	3 min at 30% MVC	Significant increase in BP and HR
Woehrle (2018) [77]	19 concussed adolescents 16 healthy controls Age: 15 ± 2 years)	30 s at 30% MVC	Greater ΔHR among control participants (13 ± 10 BPM) compared with concussed patients (6.4 ± 6.3 BPM)
Garg (2013) [79]	100 participants aged 17–24 years with or without a family history of primary hypertension	2 min at 30% MVC	Greater ΔSBP, ΔDBP and ΔMAP in offspring of hypertensive parents

*BP* blood pressure, *BPM* beats per minute, *SBP* systolic blood pressure, *DBP* diastolic blood pressure, *HR* heart rate, *MAP* mean arterial pressure, *MVC* maximum voluntary contraction, *ΔHR* difference in heart rate between rest and exercise, *ΔMAP* difference in mean arterial pressure between rest and exercise

Studies are starting to emerge focused on the effects of well-known risk factors for an impaired childhood cardiovascular development on the cardiovascular stress response to exercise. Several studies suggest that a high childhood body mass index or a family history of hypertension may lead to alterations in the cardiovascular stress response to isometric handgrip exercise, although results are still inconsistent [51, 81, 82]. A study among 27 boys with a mean age of 11 years showed a lower increase in heart rate in response to isometric handgrip exercise in obese boys than in normal weight boys [68]. Similarly, a study in 20 obese children and 20 normal weight children aged 12–16 years old, found that obese children had a higher resting diastolic blood pressure, but a lower increase in diastolic blood pressure after an isometric handgrip exercise of 30% MVC until the point of fatigue [75]. A study among 14 obese children and 14 normal weight children divided into fit or unfit subgroups according to their performance of an exercise test showed that changes in cardiac output, cardiac index and stroke volume after a 3 min handgrip exercise at 30% MVC were higher in unfit than in fit obese children [72]. Contrary, a study among 166 healthy children with a mean age of 11, found a significant increase in heart rate and blood pressure after isometric handgrip exercise, but no differences among different BMI quintiles [83]. A cross-sectional study among 100 participants aged 17–24 years showed that among adolescents with a family history of primary hypertension, systolic blood pressure, diastolic blood pressure and mean blood pressure increases to a 2 min isometric handgrip exercise at 30% MVC were much more pronounced compared to adolescent without a family history of hypertension [79]. This finding was in line with a study among 47 children aged 10 to 18 years which showed no difference in heart rates at baseline, but after an isometric handgrip of 4 min at 25% MVC heart rates were significantly higher in children with a family history of primary hypertension [74].

Thus, overall these relatively small studies suggest that isometric handgrip exercise at varying rates of intensity, induces alterations in the cardiovascular system. Most of these studies have used an exercise protocol that consisted a 3 min isometric handgrip exercise with a MVC at 30% [68–70, 72, 76, 77]. So far, small studies suggest that already subtle differences in heart rate and blood pressure response to isometric handgrip exercise may be present among higher risk pediatric populations. However, long-term follow-up studies focused on the associations of differences in cardiovascular stress response to isometric handgrip exercise in childhood with cardiovascular health outcomes in adulthood have not yet been performed.”

## Isometric Handgrip Exercise and the Effects on Cardiac Adaptations Measured by Advanced Imaging Techniques

The use of isometric handgrip exercise to induce cardiac adaptations measured during cMRI has been studied in multiple adult studies, but not yet among pediatric populations. As no pediatric studies are yet available, we reviewed the evidence from adult studies to explore the effect of isometric handgrip exercise on cardiac adaptations as part of the cardiovascular stress response. Table 3 shows a descriptive overview of all studies found by our Medline search among adult populations assessing the cardiovascular stress response on isometric handgrip exercise during a MRI scan.

**Table 3:** Descriptive overview of studies examining the effects of isometric handgrip exercise on cardiovascular outcomes in adults during MRI scans

Name, year	Population	Used handgrip exercise protocol	MRI protocol	Cardiac outcomes
Al-Otaibi (2010) [84]	One epileptic 24 year old patient 10 healthy subjects: age: 27.3 ± 4.0 years	2 exercise conditions: 30% MVC and 70% MVC. Each session consisted of repeated handgrip contractions each lasting 2 s. 27 trials were completed per condition	FMRI of the brain	The HR response to the IHE was lower in the patient during both 30% and 70% MVC (0.2 and 3.4BPM, respectively) relative to the control group (2.9 ± 1.8 and 7.3 ± 4.1 bpm, respectively)
Betim Paes (2013) [85]	28 patients with Chagas Heart Disease: age: 48 ± 11 years 8 healthy subjects: age: 29 ± 4 years	8 min	Magnetic Resonance Spectroscopy of the heart	Both groups had a significant HR and RPP increase after exercise. The control group had a higher mean HR both at rest and during exercise
Bonanno (2018) [86]	10 healthy subjects: age: 24 ± 5.5 years	5–8 min at 30% MVC	Coronary MRI	RPP increase: 37%
Globits (1997) [87]	9 healthy subjects: age: 31 ± 4 years	3 min at 50% MVC	Coronary MRI	HR increase: 24% Mean BP increase: 25% RPP increase: 54.4%
Haddock (2018) [88]	10 healthy subjects: age: 20–48 years	5-min at 70% MVC	Renal arterial flow (RAF)	HR: increase: 17 ± 9% Systolic BP increase: 25 ± 11%
Hays (2010) [59]	20 healthy subjects: age: 40 years 17 patients with CAD: age: 55 years	4,5 min at 30% MVC	Coronary MRI	Healthy: HR increase: 15.9% MAP increase: 12.5% RPP increase: 27% CAD: HR increase: 12.6% Mean BP increase: 12.5% RPP increase: 26% The RPP during IHE and the percent increase in RPP from baseline did not significantly differ between CAD patients and healthy subjects
Hays (2010) [59]	20 healthy subjects: age: 40.2 ± 13.7 years 17 patients with CAD: age: 55.5 ± 6.8 years	4.5 min 30% MVC	Coronary MRI	Healthy: HR increase: 15.9% Systolic BP increase: 12.5% RPP increase: 27% CAD: HR increase: 12.6% Systolic BP increase: 12.5% RPP increase: 26%
Hays (2012) [89]	14 healthy subjects: age: 39 ± 19 years 14 patients with non-obstructive CAD: age: 59 ± 7 years	4½ minutes at 30% MVC	Coronary MRI	Healthy: HR increase: 15.7% Systolic BP increase: 9.6% RPP increase: 28% CAD: HR increase: 17.0%, Systolic BP increase: 9.2% RPP increase: 28%
Hays (2015) [67]	10 healthy subjects: age: 31 years 8 patients with CAD: age: 60 years	30% MVC	Coronary MRI	Coronary arteries in healthy subjects significantly dilated in response to IHE. RPP increase: 8000 to 12,000
Hays (2017) [61]	29 subjects with CAD: Age: 58 years 16 healthy subjects: Age: 57 years	4,5 min at 30% MVC	Coronary MRI	Healthy: RPP increase: 30.1 ± 17.6% CAD: RPP increase: 32.8 ± 17.2% Difference between healthy and CAD was not significant
Iantorno (2016) [90]	26 healthy subjects, age: 45 ± 3.5 years 15 patients with CAD, age: 61 ± 1.5 years	4 to 7 min at 30% MVC	Coronary MRI	IHE induced significant and similar hemodynamic changes in healthy subjects and patients with CAD Healthy: RPP increase: 35.4 ± 4.6% CAD: RPP increase: 28.7 ± 3.9%
Iantorno (2017) [91]	18 patients HIV + CAD-, age: 52 years 36 patients HIV- CAD-, age: 52 years 41 patients HIV- CAD + , age: 59 years 17 patients HIV + CAD + , age: 59 years	4–7 min at 30% MVC	Coronary MRI	HIV + patients with no significant CAD have severely impaired CEF that is similar to that of HIV- patients with established CAD.No significant differences in mean RPP change or peak RPP during IHE among the four groups
Iantorno (2018) [92]	36 patients HIV + CAD-: age: 53 ± 8 years 15 patients HIV + CAD + : age: 57 ± 4 years 14 patients HIV-CAD-: age: 50 ± 7 years	6–7 min at 30% MVC	Coronary MRI	HIV + CAD-: RPP increase: 17% HIV + CAD + : RPP increase: 21% HIV-CAD-: RPP increase: 25%
Knobelsdorff-Brenkenhoff (2016) [60]	7 patients with hypertensive heart disease [HYP]:age: 56 ± 12 years 12 patients with aortic stenosis [AS]: age: 60 ± 15 years 24 healthy subjects: age: 47 ± 17 years	6–8 min at 30% MVC	Heart protocol	HYP subjects showed a higher systolic blood pressure during exercise than controls HYP HR increase: 20.6612.1% Systolic BP increase: 19.469.0% AS HR increase: 12.566.6% Systolic BP increase: 16.4618.9% Healthy HR increase: 15.368.5% Systolic BP increase: 13.169.2%
Knobelsdorff-Brenkenhoff (2013) [52]	53 healthy subjects: age: 45 ± 17 years	6–8 min at 30% MVC	Heart protocol	HR increase: 20 ± 13%, Systolic BP increase: 15 ± 11%: Diastolic BP increase: 20 ± 18% Mean BP increase: 17 ± 13%, RPP increase: 37 ± 21%, CO increase: 27 ± 16% Stroke volume did not significantly increase. Higher age was associated with reduced increase of stroke volume and cardiac output Overweight subjects showed less increases in heart rate and cardiac output
Leucker (2018) [93]	48 HIV + patients: age: 49 ± 8 years 15 healthy subjects: age: 52 ± 12 years	4 to 7 min at 30% MVC	Coronary MRI	CEF was significantly reduced in the HIV + versus HIV- subjects
Macey (2017) [94]	63 healthy subjects: age: 47.0 ± 9.1 years	4 × 16 s challenges at 80% MVC	FMRI of the brain	Females showed higher resting HR than males, but smaller percent HR change increases during exercise
Mathews (2017) [95]	30 healthy women: age: 49.8 ± 16.7 years 20 healthy men: age: 44.1 ± 16.4 years	5–6 min at 30% MVC	Coronary MRI	In men baseline CSA was 13.4 ± 4.6 mm^2^ and increased 8.8 ± 5.2% with IHE. In women baseline CSA was 10.7 ± 2.6mm^2^, and increased 1.4 ± 9.6% with IHE Men: HR increase: 20,0% Systolic BP increase: 10,7% Diastolic BP increase: 15,9%: RPP increase: 33,9% Women: HR increase: 17,2% Systolic BP increase: 8,0% Diastolic BP increase: 17,9% RPP increase: 28,1%
Norton (2013) [96]	29 subjects: age: 21–80 years	40% MVC	FMRI of the brain	The average change in HR from baseline was 6BPM
Norton (2015) [97]	23 healthy subjects: age: 63 years 17 patients with CAD: age: 59 years	7 repeated bouts at 40% MVC with each contraction lasting 20 s and separated by 40 s of rest	FMRI of the brain	HR during exercise in control participants was greater than CAD patients Specifically, young individuals (25 ± 4 year) have a larger HR response (6–15 beats/min) to a similar relative IHE tension
Rokamp (2014) [98]	11 healthy subjects: age: 24 ± 3 years	Squeeze 30–60 times per minute with as much effort as possible	FMRI of the brain	Diastolic BP increase: 4 mmHg Mean BP increase: 5 mmHg No significant changes were observed for SBP and HR
Verbree (2017) [99]	20 healthy subjects: age: 30 years	The first minute at 80% MVC to be directly followed by 4 min at 60% MVC	Middle cerebral artery	HR increase: 11.2 ± 1.7%
Williamson (2003) [100]	8 healthy subjects: age: 26 ± 3 years	IHE beginning at 40% MVC until 15 mmHg BP increase	FMRI of the brain	Mean BP increase: 14,9% HR increase: 7 ± 3 BPM
Wong (2007) [101]	17 healthy subjects: age: 25 ± 4 years	3 × 30 s blocks separated by 1 min of rest at 5% or 35% MVC	FMRI of the brain	HR and MAP were increased in the 35% MVC trials but not the 5%MVC trials. Both the left and right hand trials elicited similar cardiovascular responses
Wood (2017) [102]	52 healthy subjects Age: 59 years	7 repeated bouts at 40% MVC. Each contraction bout lasted 20 s and was separated by 4 s of rest	FMRI of the brain	HR responses to IHE showed high variability across individuals. Linear regression revealed that cardiorespiratory fitness was not a strong predictor of the HR response
Zhang (2012) [103]	4 healthy subjects Age: 25–36 years	3 × 1 min at 100%MVC	Retina/choroid blood flow	HR increase: 19% ± 8%, Mean BP increase: 22% ± 5%

No comparable pediatric studies are available

*BP* blood pressure, *BPM* beats per minute, *CAD* coronary artery disease, *CBF* coronary blood flow, *CEF* coronary endothelial function, *CSA* cross-sectional area, *DBP* diastolic blood pressure, *FMRI* functional magnetic resonance imaging, *HR* heart rate, *IHE* isometric handgrip exercise, *MAP* mean arterial pressure, *MRI* magnetic resonance imaging, *MVC* maximum voluntary contraction, *RPP* Rate pressure product = Heart Rate*Systolic Blood Pressure, *SBP* systolic blood pressure

The majority of these studies examined the effects of a sustained handgrip at 30% MVC for 3–8 min during the MRI scanning. Even though isometric handgrip exercise protocols performed in the MRI varied, most studies showed that heart rate, systolic and diastolic blood pressure, rate pressure product (heart rate*systolic blood pressure), cardiac output and left ventricular ejection fraction significantly increased during exercise in line with observed responses among pediatric populations. A study in 53 healthy subjects (age 35 ± 17 years) used an isometric handgrip protocol of 6–9 min of sustained contraction at 30% MVC and showed that stroke volume and CO (L/min) increased. Overweight subjects showed less increase in heart rate and cardiac output [52]. This is in accordance with a study done in 75 healthy volunteers (age 38.8 ± 10.9 years) that examined the effects of biceps isometric exercise and found that BMI is associated with reduced augmentation of the CO [35]. Isometric handgrip exercise during cMRI can also be used to examine coronary endothelial function (CEF) [59, 61, 62, 67, 86, 87, 89–91, 93, 95, 104–106]. Healthy coronaries respond to exercise with a release of nitric oxide which lead to vasodilatation and an increase in coronary blood flow. Abnormal endothelial nitric oxide release leads to paradoxical vasoconstriction and reduced coronary blood flow which is an indicator of early atherosclerosis and a predictor of future disease [62, 67, 89, 105, 106]. A study in 14 healthy adults and 14 adult patients with non-obstructive mild coronary artery disease (< 30% maximum stenosis) examined the effects of isometric handgrip exercise of 4.5 min at 30% MVC on CEF. The coronary vasoreactivity (percentage change in coronary cross-sectional area) to isometric handgrip exercise was significantly higher in healthy subjects (13.5 ± 12.8%) than in those with mild coronary artery disease (− 2.2 ± 6.8%, p < 0.0001) [89].

Thus, these results show that isometric handgrip exercise can be performed successfully during cMRI in adult populations. An exercise protocol consisting a sustained handgrip at 30% MVC for 3–8 min results in significant hemodynamic changes that has the potential to reveal subtle functional cardiac differences in cMRI measurements. Cardiac adaptations as part of the cardiovascular stress response on handgrip exercise examined by cMRI have yet to be explored within pediatric populations.

## Further Research

Accumulating evidence suggests that cardiovascular diseases may at least partly originate from early life onwards. However, the mechanisms underlying the observations that early life is a critical period for cardiovascular health in later life remain unclear. Also, early identification of children at risk of reduced cardiovascular health in adulthood remains challenging. In adults, the cardiovascular exercise stress test is already more commonly used in clinical and research settings to reveal subtle cardiovascular differences among individuals at risk for cardiovascular pathology. Based on this narrative review, we showed for the first time that a cardiovascular exercise stress test through a simple handgrip exercise may also have additional value as a marker of a suboptimal cardiovascular health profile in pediatric populations. Although many different handgrip exercise protocols exist, based on our narrative review it seems that a sustained handgrip at 30% MVC for 3–4 min is already sufficient to significantly raise blood pressure and heart rate in children and reveal differences in the cardiovascular stress response in children with cardiovascular risk factors, e.g., obesity as compared with healthy children. Thus, assessment of the cardiovascular stress response to relatively light handgrip exercise may be a novel method to already detect subtle differences in cardiovascular health from early childhood onwards. This method may provide novel insight into underlying mechanisms and may aid in earlier identification of children at higher risk of cardiovascular disease in later life. Yet, there remain important issues to be addressed.

First, thus far only small studies have examined the effects of isometric handgrip exercise on the cardiovascular stress response in non-diseased children. These studies have focused on heart rate and blood pressure variability in response to isometric handgrip exercise. None of these studies used advanced imaging techniques to assess cardiac adaptions in response to isometric handgrip exercise. Further research is needed to assess the detailed cardiovascular effects of isometric handgrip exercise in children using a combination of simple clinical measurements and advanced imaging techniques and to assess the feasibility of these measurements within large population studies from early childhood onwards. It further remains to be established whether isometric handgrip exercise is the most feasible method in pediatric population research to induce to cardiovascular stress response to exercise or whether a more high-intensity exercise method is needed to induce a clinically relevant cardiovascular stress response. Studies comparing different exercise methods in combination with detailed cardiovascular measurements in pediatric populations are needed.

Second, studies are needed to explore the associations of well-known cardiovascular risk factors with the cardiovascular stress response throughout childhood and adolescence into adulthood. Thus far, studies have only focused on obesity and family history of hypertension as adverse exposures leading to subclinical differences in the cardiovascular stress response. Even though these studies suggest small differences in cardiovascular stress response are present, these studies were small and show conflicting results. Further studies are needed to replicate these findings within larger pediatric populations. Also, studies are needed to explore the influence of other well-known cardiovascular risk factors, already from early fetal life onwards, on the cardiovascular stress response, such as maternal obesity during pregnancy, preterm birth and low birth weight.

Finally, long-term follow-up of participants is needed to obtain insight into the cardiovascular consequences later in life of an abnormal cardiovascular stress response in childhood and to explore whether the assessment of the cardiovascular stress response is beneficial for screening for individuals at a higher risk of cardiovascular disease in later life.

## Conclusion

Cardiovascular diseases are a major public health problem with a large impact on morbidity and mortality rates worldwide. Accumulating evidence suggests that cardiovascular diseases may at least partly originate in the earliest phase of life. Adverse exposures in early life may lead to permanent adaptations in the cardiovascular system, predisposing to cardiovascular diseases in later life. The cardiovascular stress response to exercise may be a valuable additional measurement to detect subtle differences in cardiovascular health already from early childhood onwards. Based on small studies in pediatric and adult diseased and non-diseased populations, measurement of simple clinical measures including heart rate and blood pressure variability in combination with advanced imaging techniques to assess detailed cardiac adaptations in response to isometric handgrip exercise, can reveal subtle differences in cardiovascular development, which are associated with short-term and long-term cardiovascular health outcomes. Well-designed epidemiological studies from early childhood onwards are needed to assess the use and feasibility of measuring the cardiovascular stress response to exercise as a novel marker of cardiovascular health. These studies need to focus on the influence of well-known risk factors from early life onwards for cardiovascular disease on cardiovascular stress response in childhood and adolescence and assess whether differences in the cardiovascular stress response throughout childhood and adolescence are associated with cardiovascular health outcomes in later life.
